# Systematic analysis of relationships between plasma branched-chain amino acid concentrations and cardiometabolic parameters: an association and Mendelian randomization study

**DOI:** 10.1186/s12916-022-02688-4

**Published:** 2022-12-15

**Authors:** Marwah Doestzada, Daria V. Zhernakova, Inge C. L. van den Munckhof, Daoming Wang, Alexander Kurilshikov, Lianmin Chen, Vincent W. Bloks, Martijn van Faassen, Joost H. W. Rutten, Leo A. B. Joosten, Mihai G. Netea, Cisca Wijmenga, Niels P. Riksen, Alexandra Zhernakova, Folkert Kuipers, Jingyuan Fu

**Affiliations:** 1 Department of Genetics, University Medical Center Groningen, University of Groningen, Groningen, the Netherlands; 2 Department of Pediatrics, University Medical Center Groningen, University of Groningen, Groningen, the Netherlands; 3 Laboratory of Genomic Diversity, Center for Computer Technologies, ITMO University, St. Petersburg, Russia; 4 Department of Internal Medicine and Radboud Center for Infectious Diseases, Radboud University Medical Center, Nijmegen, the Netherlands; 5 Department for Genomics Immunoregulation, Life and Medical Sciences Institute, University of Bonn, Bonn, Germany; 6 Human Genomics Laboratory, Craiova University of Medicine and Pharmacy, Craiova, Romania; 7 University of Groningen, University Medical Center Groningen, European Institute of Healthy Ageing (ERIBA), Groningen, the Netherlands

**Keywords:** Branched-chain amino acids, Cardiometabolic diseases, Population-based studies, Mendelian randomization

## Abstract

**Background:**

Branched-chain amino acids (BCAAs; valine, leucine, and isoleucine) are essential amino acids that are associated with an increased risk of cardiometabolic diseases (CMD). However, there are still only limited insights into potential direct associations between BCAAs and a wide range of CMD parameters, especially those remaining after correcting for covariates and underlying causal relationships.

**Methods:**

To shed light on these relationships, we systematically characterized the associations between plasma BCAA concentrations and a large panel of 537 CMD parameters (including atherosclerosis-related parameters, fat distribution, plasma cytokine concentrations and cell counts, circulating concentrations of cardiovascular-related proteins and plasma metabolites) in 1400 individuals from the Dutch population cohort LifeLines DEEP and 294 overweight individuals from the 300OB cohort. After correcting for age, sex, and BMI, we assessed associations between individual BCAAs and CMD parameters. We further assessed the underlying causality using Mendelian randomization.

**Results:**

A total of 838 significant associations were detected for 409 CMD parameters. BCAAs showed both common and specific associations, with the most specific associations being detected for isoleucine. Further, we found that obesity status substantially affected the strength and direction of associations for valine, which cannot be corrected for using BMI as a covariate. Subsequent univariable Mendelian randomization (UVMR), after removing BMI-associated SNPs, identified seven significant causal relationships from four CMD traits to BCAA levels, mostly for diabetes-related parameters. However, no causal effects of BCAAs on CMD parameters were supported.

**Conclusions:**

Our cross-sectional association study reports a large number of associations between BCAAs and CMD parameters. Our results highlight some specific associations for isoleucine, as well as obesity-specific effects for valine. MR-based causality analysis suggests that altered BCAA levels can be a consequence of diabetes and alteration in lipid metabolism. We found no MR evidence to support a causal role for BCAAs in CMD. These findings provide evidence to (re)evaluate the clinical importance of individual BCAAs in CMD diagnosis, prevention, and treatment.

**Supplementary information:**

The online version contains supplementary material available at 10.1186/s12916-022-02688-4.

## Background

The worldwide obesity epidemic represents a major health burden, with obesity leading to a spectrum of comorbidities that are the basis for cardiometabolic diseases (CMD). CMD are multifactorial metabolic diseases that can include insulin resistance (IR), type 2 diabetes (T2D), and cardiovascular disease (CVD) [1, 2], with other conditions like hyperlipidemia and hypertension also related to CMD. Circulating concentrations of the branched-chain amino acids (BCAAs), isoleucine, leucine, and valine have consistently been associated with CMD and CMD risk factors in various studies [3–5]. BCAAs are essential amino acids strictly sourced from diet that have several physiological and metabolic roles and have been established to be risk factors for IR and diabetes [6–8]. Recent literature, however, has challenged the role of BCAAs as mere risk factors by suggesting that they also play an etiological role in CMD development. For IR and diabetes, multiple prospective and mechanistic studies have now produced evidence for BCAAs playing a causal role. For instance, in a prospective cohort of 6244 individuals, higher baseline concentrations of BCAAs were associated with IR and found to predict incident T2D in a 7.5-year follow-up, independent of other risk factors [9]. Similarly, higher concentrations of BCAAs also predicted the development of diabetes in women with a history of gestational diabetes and IR [10], even in young non-glycemic adults [11].

Mechanistic studies using mouse models have shed light on the pathways potentially involved. One leading hypothesis suggests that BCAAs, or their breakdown products (branched-chain keto acids), have a direct influence on key factors involved in the pathogenesis of diabetes through their interaction with the mammalian target of rapamycin (mTOR) signaling pathway [3, 12] and through induction of oxidative stress [13–16], mitochondrial dysfunction [17, 18], apoptosis [19, 20], and IR and/or impaired glucose metabolism [3, 21–28]. In line with this, White et al. were the first to show that BCAA supplementation to a high fat diet downregulated the AKT pathway (pAKT), a marker of insulin signaling in muscle, leading to impaired glucose tolerance via hyperactivation of mTOR signaling in muscle [29]. This finding is further supported by a study showing that BCAA metabolites downregulated pAKT [25] and by other studies showing that BCAA supplementation with a high fat diet or defective BCAA oxidation in mice induces IR [3, 26].

Relationships between BCAAs and other CMD traits are also emerging, but it is still unclear whether they represent important indicators of CMD risk or even play a causal role. In this regard, some of the most extensively studied CMD traits are CVDs, a set of diseases including atherosclerosis and myocardial infarction, that affect the heart and the circulatory system. Several studies demonstrated that increased concentrations of various BCAAs were associated with increased CVD risk [30] (e.g., for hypertension [31]) and that their presence in plasma is an independent predictor for adverse cardiovascular events [32]. Yet, the BCAA–CVD nexus is not as straightforward for other CVDs and clinical contexts, e.g., for atherosclerosis. While a strong correlation between atherosclerosis and BCAAs has been reported, the association was not as clear in obese individuals [33]. Indeed, the influence of BCAAs in CVD in obese subjects, as well as their role in the severity of obesity, has yet to be properly understood. This is mostly because atherosclerosis and body fat distribution are closely related to CVD, but there is also mounting evidence that fat-tissue metabolism is key in determining blood concentrations of BCAAs [34, 35].

One way to better understand the correlation between BCAAs and CMD would be to look at a wide range of CMD-related parameters, including CVD risk factors [36]. The OLINK panel provides a large toolkit of known CVD-related proteins and could potentially unravel a correlation of BCAAs and CMD [37]. Several other emerging CVD biomarkers, such as trimethylamine N-oxide (TMAO) and its precursors, should also be considered for this screening given their recently confirmed correlation in T2D patients [38–40]. Likewise, several other important components of CMD, such as lipid metabolism, inflammation, and immunity, are also potential biomarkers, but their relationships with BCAAs are poorly understood. The interplay between BCAAs and lipids in the onset of various CMD, such as IR and chronic hyperinsulinemia, has been widely suggested [3, 25, 26, 41, 42], but the relationship between BCAAs and lipids has not been systematically explored, even though it could shed light on a common pathway to CMD. Similarly, BCAAs can pose as donors of nitrogen and carbon skeletons for the synthesis of other amino acids that have major roles in immune cell function [43]. BCAAs have also been shown to activate inflammatory signals and increase the release of inflammatory cytokines, which exacerbates IR by blocking insulin signaling transduction in adipocytes and skeletal muscle cells [44]. Still, as a nascent field of research, many aspects of BCAAs and their effects on inflammation and immune function, along with their concomitant role in CMD, are poorly explored, and a first step toward resolving this knowledge gap would be to systematically explore the relation of BCAAs and inflammatory and immune parameters in large human cohorts.

Taken together, the relationship between BCAAs and some components of CMD, such as IR/diabetes, have been extensively studied, but the relationship of BCAAs and other CMD parameters has yet to be established, as most studies to date are either pre-clinical or performed for sex- and disease-specific conditions. In addition, while many of the mechanistic studies in animal models have provided evidence of individual-level causality, systematic evaluation in human cohorts is crucial to provide population-level evidence and identify the potential etiological roles of BCAAs in CMD. Moreover, previous results about the direction of the causal relationship between BCAAs and CMD are conflicting. For example, a causal role for BCAAs in IR was supported in a study by Lotta et al. [45], while reverse causality of IR on BCAAs has been suggested by other studies [46, 47]. Here, we present a systematic, cross-sectional association analysis between fasting plasma concentrations of BCAAs and a large panel of 537 parameters (including clinical CMD measures such as non-invasive measures of atherosclerosis, fat distribution, circulating CVD-related proteins, plasma metabolites and inflammatory cell counts and immune cytokines) in 1400 individuals from the general population–based LifeLines DEEP (LLD) cohort [48] and 294 overweight/obese individuals from 300OB the cohort [49]. In this study, we (1) establish association relationships between BCAAs and CMD-related traits that are independent of age, sex, BMI, and other potential covariates; (2) estimate and compare the association strength between different BCAAs; and (3) interrogate the potential causal direction of associations using a bi-directional Mendelian randomization (MR) approach.

## Methods

### Cohort description

#### LifeLines DEEP

The LifeLines study is a large prospective, population-based cohort study from the north of the Netherlands. This study started in 2006 and aims to follow 167,729 participants for 30 years in order to identify the biomedical, socio-demographic, behavioral, physical, and psychological factors that contribute to health and disease in the general Dutch population [48]. The study includes a three-generation design. Individuals aged 25–50 years and their family members (partner, parents, and children) were invited by their general practitioner to participate in the LifeLines study. The study employs a broad range of phenotypic measures and questionnaires. At baseline, all participants filled in two extensive baseline questionnaires at home and then twice visited one of the LifeLines research sites for physical examinations. At the first visit, anthropometry, blood pressure, cognitive functioning, and pulmonary function, as well as other factors, were measured. At the second visit, approximately 2 weeks later, a fasting blood sample was collected. Afterwards, each participant receives a follow-up questionnaire every 18 months. Follow-up measurements of the health parameters are performed every 5 years.

LifeLines DEEP (LLD) is a sub-cohort of the LifeLines cohort that consists of 1539 individuals. From April to August 2013, all participants registered at the LifeLines research site in Groningen were invited to participate in the LifeLines DEEP study, a study with deep omics profiling in addition to the regular LifeLines program. Eventually, 1539 randomly selected individuals participated in LLD. For these participants, extra biomaterials (plasma, exhaled air and feces) were collected for various omics profiling, such as genetics, methylation, transcriptomics, metabolomics, proteomics, and microbiome. A detailed cohort description can be found in Tigchelaar et al. [48] and Zhernakova et al. [50]. For the current study, we included 1400 LLD participants after excluding individuals with missing information for most omics data and 23 diabetes patients. A total of 537 CMD-related phenotypes were assessed in our study. Due to different missing values for each dataset, the number of individuals analyzed differs as shown in Additional file 1: Table S1. The method description of datasets is also listed below.

#### 300OB

300OB is an obese cohort of 302 individuals aged 54–81 years old, who were enrolled in the study at the Radboud University Medical Center, Nijmegen, the Netherlands. A detailed description is provided in our earlier paper [49]. In brief, all participants had a body mass index (BMI) ≥ 27 kg/m^2^ at screening (mean = 30.73, median = 29.89). Exclusion criteria were a recent cardiovascular event (myocardial infarction, transient ischemic attack or stroke < 6 months), a history of bariatric surgery or bowel resection, inflammatory bowel disease, renal dysfunction, increased bleeding tendency, use of oral or subcutaneous anti-coagulant therapy, use of thrombocyte aggregation inhibitors other than acetylsalicylic acid and carbasalate calcium (antithrombotic), or a contra-indication for magnetic resonance imaging (MRI). Participants who used lipid-lowering therapy temporarily discontinued this medication 4 weeks prior to the measurements. All women were postmenopausal and did not use hormonal replacement therapy. For all participants, blood samples for nuclear magnetic resonance (NMR)-based lipidomics, cytokines and cell counts, TMAO measures, and OLINK panel III CVD measurements were collected in the morning following an overnight fast. In the present study, we included 294 participants for whom fasting plasma metabolites were available. Due to different missing values for each dataset, the number of individuals analyzed differs as shown in Additional file 1: Table S1.

For both cohorts, multiple concentrations of omics data are available. In this study, we used the detailed phenotypic data routinely used in clinic to assess CVD risk, non-invasive measures of fat distribution and carotid intima-media thickness (IMT) as a non-invasive measure of atherosclerosis, metabolomics (mainly including lipoproteins), TMAO and related metabolites, circulating proteins, and cell counts and cytokines.

### Cardiovascular phenotyping in the 300OB cohort

In 300OB, fat distribution was assessed using MRI, including volumes of visceral adipose tissue and subcutaneous adipose tissue, divided into deep and superficial subcutaneous adipose tissue, respectively. Hepatic fat content was quantified using localized proton magnetic resonance spectroscopy. Non-invasive measures of atherosclerosis of the carotid arteries included measurement of carotid IMT, carotid plaque presence and maximum plaque thickness, and plaque presence and maximum thickness in the common carotid, internal carotid, external carotid artery, and carotid bulbus. The presence of plaque was defined as focal thickening of the wall of at least 1.5 × mean IMT or an IMT > 1.5 mm, according to the Mannheim IMT consensus [51]. All measurements were performed at Radboud University Medical Center. Further details are described in a previous paper [49].

### OLINK circulating proteins

Blood samples of participants from both cohorts were collected separately and frozen at − 80 °C prior to any measurement. For both cohorts, circulating proteins from EDTA plasma samples were measured using commercially available OLINK Proteomics (Uppsala Sweden), the AB Inflammation Panel for 300OB (162 CVD II panel and inflammation panel), and OLINK Proseek Multiplex CVD III panel for LLD (92 CVD proteins). OLINK provides a multiplex immunoassay for high-throughput detection of protein biomarkers in liquid samples. Proteins are recognized by antibody pairs coupled to cDNA strands that bind in close proximity and extend by a polymerase reaction. In 300OB, proteins were excluded from analysis when the detection level of 75% was not met. Quality control (QC) was performed by OLINK Proteomics, with two samples that did not pass QC subsequently excluded from the analysis. OLINK panel data was available for 1294 participants from LLD and 294 samples from 300OB. More detailed information can be found at the OLINK site: http://www.olink.com/products.

### NMR

For both cohorts, we profiled a wide range of plasma metabolites using NMR and the Nightingale Biomarker Analysis Platform [52]. This platform provides a wide range of plasma metabolites including lipid concentrations; relative compositions of 14 lipoprotein subclasses; lipoprotein particle sizes and concentrations of apolipoproteins; cholesterol; triglycerides, and phospholipids; and several glycolysis components, fatty acids, inflammation markers, ketone bodies, and amino acids. This platform provides measures of 231 plasma metabolome traits, which included the three BCAA, in both cohorts. For the other traits, we included 228 metabolites in LLD but only 223 metabolites in 300OB due to a large percentage of missing values for five measurements. Our group had previously validated platform precision by comparing several traits and observed a high degree of consistency [49]. All the NMR measurements in LLD samples were performed in one batch, whereas 300OB samples were randomized and measured in two batches. Therefore, for association analyses in the 300OB cohort data, batch number was included as an additional covariate.

### TMAO, choline, betaine and γ-butyrobetaine profiling

We used ultra-high-performance liquid chromatography in combination with isotope dilution tandem mass spectrometry to analyze TMAO, choline, L-carnitine, and betaine for both cohorts and γ-butyrobetaine in plasma specifically in the 300OB cohort, as described elsewhere [49].

### Cell counts and cytokine profiling

Blood cell counts were measured for both cohorts, and we used six cell types related to immunity for further analysis: basophils, eosinophils, granulocytes, lymphocytes, monocytes, and thrombocytes. The cytokines for the LLD cohorts included IL (interleukin)-1β, IL-6, IL-8, IL-10, IL-12p70, and tumor necrosis factor-α (TNF-α) measured by ProcartaPlex multiplex immunoassay (eBioscience, San Diego, CA). Other inflammation markers, including leptin, adiponectin, IL-18, IL-18BP, and resistin, were measured using commercially available sandwich ELISA kits (R&D Systems, Minneapolis, MN). For the 300OB cohort, measured cytokines included IL-1β, IL-6, IL-18 (including IL-18BP), and inflammation markers included adiponectin, leptin, and resistin, all measured according to protocols described earlier [49].

### Association analyses

First, we identified age, sex, and BMI as important covariates in assessing the association between BCAAs and CMD parameters. Next, we explored whether other lifestyle and dietary factors can be potential confounders. If a factor is an important confounder, we expected to see a significant association between this factor and the exposure, i.e., BCAAs. We therefore made use of the LLD cohort to assess the associations between BCAAs and all potential lifestyle and dietary covariates using a univariable regression model. The lifestyle and dietary factors assessed included current smoking status, intake of alcohol, meat, fruit, vegetables, and coffee and total energy intake in kcal and macronutrients (carbohydrates, total protein, animal protein, plant protein, and fat) (Additional file 1: Table S2). These lifestyle factors were obtained from questionnaires filled in by each participant. Dietary intake was assessed using a semi-quantitative Food Frequency Questionnaire (FFQ) [48]. While we did observe strong associations with age, sex, and BMI, we did not observe any significant associations with smoking or dietary factors (Additional file 1: Table S2). This led us to conclude that the potential confounding effects of these factors were negligible, if present at all. We therefore only considered age, sex, and BMI as important covariates and assessed pair-wise associations between individual BCAAs and different CMD traits using linear regression models where *phenotype* ~ *BCAA* + *age* + *sex* + *BMI.* The independent and continuous dependent variables were scaled using a z-scale transformation (to obtain values ranging from − 1 to 1) to be able to compare the effect size of different categories and in both cohorts. Logistic regression was used for binary phenotypes. Each BCAA was analyzed separately without mutually adjusting for the others.

Multiple regression requires the data to fulfill several assumptions: the response variable is assumed to be a linear function of the model parameters (1) and model errors are assumed to be independent (2), to have a constant variance (3), and to be normally distributed (4). In addition, the predictors are assumed to be measured without error (5), and there are assumed to be no unmeasured confounders that affect both the response and predictor variables (6). To ensure that major assumptions 1–4 were fulfilled, we manually inspected diagnostic plots of the models for each BCAA–phenotype combination. To conform with the normality assumption, we log-transformed 27 phenotypes (see Additional file 1: Table S1). Assumptions 5 and 6 could not be directly assessed, which is one of the limitations of our study.

We note that we included BMI as a covariate because its causal role in BCAAs and cardiometabolic traits has been widely studied. In this study, we wanted to correct for the confounding effects of BMI so we could investigate the associations of BCAAs with various CMD-related parameters, including fat distribution, independent from overall obesity as represented by BMI. NMR measurements in the 300OB cohort were also adjusted for batch number. Where applicable, we included additional covariates, including protein and meat intake and HOMA-IR for NMR analysis in LLD, as mentioned in the “Results” section. In addition, BCAAs have been implicated in kidney function, which is also linked to cardiometabolic health. We therefore also checked the LLD cohort for associations between BCAAs and kidney function, which was assessed by estimated glomerular filtration rate (eGFR). The latter was calculated using the original Modification of Diet in Renal Disease formula. All three BCAAs were significantly associated with eGFR (Additional file 1: Table S2). As eGFR data was not available for the 300OB cohort, we did not include eGFR as a covariate but did compare results with or without eGFR as a covariate in the LLD cohort. To correct for multiple testing, *P* values were further adjusted for 537 traits × 3 BCAA for each cohort using the Benjamini–Hochberg method, with significance set at a false discovery rate (FDR) < 0.05. These are reported as the FDR-value in both the tables and in the Results section where appropriate. All analyses were performed using R version 3.61.

### Mendelian randomization

To determine causality between BCAAs and associated factors, two-sample bi-directional MR was performed using the R package TwoSampleMR v.0.5.6 with default settings [53]. The genetic variants used as instrumental variables for MR analyses and their effect sizes were obtained from publicly available summary statistics for genome-wide association studies (GWASs) on BCAAs and cardiometabolic traits. BCAA-associated SNPs were taken from the largest GWAS on metabolites to date performed in the UK Biobank and available in the OpenGWAS database [54] under accession IDs met-d-Val, met-d-Ile, and met-d-Leu. Summary statistics for all of the cardiometabolic traits were obtained from the OpenGWAS database, where we aimed recover data from the GWAS with the highest sample size, preferably based on European samples, for each CMD parameter tested in the association analysis. Genetic variants were clumped using *r*^2^ < 0.001 in 1000G EUR samples. Proxies were added automatically by the TwoSampleMR R package. To estimate the causal effect of BCAAs on CMD parameters, we used SNPs associated with the sum of all three BCAAs (accession id met-d-Total_BCAA) to overcome the potential confounding effect of the other two BCAAs when using separate BCAAs as the exposure.

To calculate univariable MR (UVMR) estimates, we used Wald ratios meta-analyzed by the inverse variance weighted (IVW) method [55]. Instrumental variables (IVs) used in MR need to fulfill three major assumptions: (1) the IVs should be associated with the exposure, (2) the IVs should not share a common cause with the outcome, and (3) the IVs should affect the outcome only through the exposure. To reduce the chances of violating these assumptions, extensive QC and sensitivity analyses were performed on the candidate MR results. In detail, results were only considered when they met the following criteria: (1) MR results were based on three or more SNPs, as this allowed us to perform the sensitivity analyses listed below; (2) MR results showed a Benjamini-Hochberg-corrected *p* value < 0.05 using IVW and nominally significant results (*p* value < 0.05) using two other MR approaches (weighted median and MR PRESSO test [56, 57]); (3) MR results did not show indications of horizontal pleiotropy or heterogeneity, as estimated using MR Egger [57] (intercept *p* value > 0.05) and the MR PRESSO [56] outlier-adjusted test (*p* value < 0.05) that estimates the pleiotropy and tries to correct for it by removing outliers; (4) MR results were not driven by single SNPs, as tested using leave-one-out analyses (no SNP after exclusion resulting in IVW MR *p* value > 0.05); or (5) genetic instruments were strong as estimated using *F*-statistics (*F* > 10). We also estimated heterogeneity using Cochran’s *Q*-test but did not filter out the results based on this measure.

BMI has previously been shown to affect both BCAA levels and CMD parameters. Moreover, BMI, BCAAs and CMD parameters have shared associated genetic variants. To overcome the potential violation of the 3rd MR assumption in the UVMR analyses, we removed BMI-associated SNPs published by the GIANT consortium [58] from the list of genetic variants. In addition, we corrected for the effect of BMI using multivariable MR analyses (MVMR). In the MVMR analysis estimating the effect of CMD parameters on BCAAs, we used two exposures: the current CMD parameter and BMI (using summary statistics published by the GIANT consortium [58]). In the MVMR in the direction from BCAAs to CMD parameters, we used four exposures: valine, isoleucine, leucine, and BMI. Sensitivity analyses of the MVMR results were performed by the MVMR v. 0.3 R package [59], which estimates the strength of the genetic instruments (*F*-statistics, which we required to be > 10) and heterogeneity using Cochran's *Q*-test.

We performed the analyses in both directions and corrected for multiple testing using the Benjamini–Hochberg method separately for each group of phenotypes (general cardiometabolic traits and NMR lipoproteins) and for each direction.

## Results

### BCAAs are widely associated with CMD parameters

**Fig. 1:**
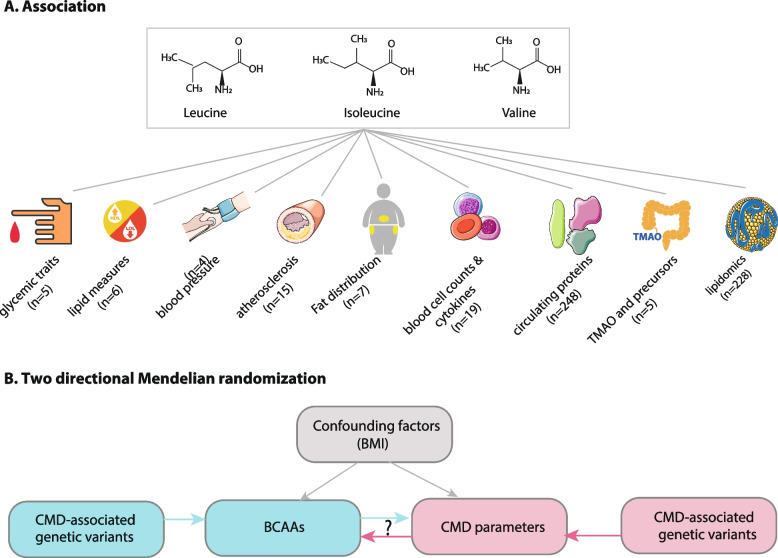
Schematic overview of the study. **A** The analysis scheme for the association analysis between the 3 branched amino acids (BCAAs) and 537 cardiometabolic disease (CMD) parameters, which are grouped into the nine categories indicated at the bottom of the panel. The number of the parameters per category is shown in parentheses. **B** The bi-directional Mendelian randomization analysis scheme. The causal relationship from BCAAs to CMD parameters is shown by the blue arrow and the causal relationship from CMD parameters to BCAAs is shown using red arrows. The impact of confounding factors such as BMI is shown by gray arrows

The basic characteristics of the LLD (*n* = 1400) and 300OB (*n* = 294) cohorts and their respective fasting plasma BCAA concentrations are summarized in Additional file 1: Table S1. The average plasma levels of BCAAs in the population-based LLD cohort were 44.9 μM for isoleucine, 57.9 μM for leucine, and 149 μM for valine. In the obesity cohort (300OB), the isoleucine concentration was 6.6 μM higher (*p* = 5.68 × 10^−8^), while the average concentrations of leucine and valine were 3.1 μM (*p* = 5.16 × 10^−5^) and 7 μM (*p* = 0.0017) lower, respectively, compared to the LLD cohort (Additional file 1: Table S1). In addition, BCAA concentrations were generally higher in males than in females and correlated positively with BMI (Additional file 1: Table S2). After correcting for age, sex, and BMI, we performed an association analysis of BCAAs with 537 CMD parameters available in either cohort, including 5 glycemic traits, 6 routine lipid measures, 4 blood pressure measures, 15 atherosclerosis-related parameters, 7 detailed fat distribution parameters, 19 cell counts and cytokines, plasma concentrations of 248 circulating proteins, 5 parameters of TMAO and its precursors, and 228 plasma metabolites (predominantly lipidomic traits) (Fig. 1, Additional file 1: Table S1).

Overall, we detected 838 significant associations for 409 unique CMD parameters in at least one of the cohorts at FDR < 0.05 (Additional file 1: Table S3). In addition, we looked at the confounding effect of kidney function, assessed by eGFR, on the associations between BCAAs and CVD in the LLD cohort. Despite being significantly associated with all three BCAA levels (Additional file 1: Table S2), correcting for eGFR did not influence the association results (Additional file 1: Table S4, Additional file 2: Figure S1).

### Isoleucine shows specific associations with lipidomic traits

**Fig. 2:**
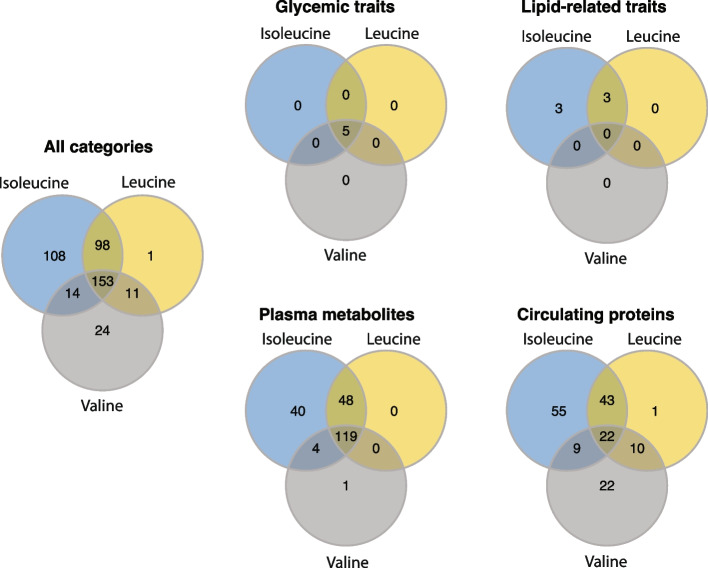
Number of CMD parameters shared between BCAAs. Venn diagrams show the numbers of CMD parameters, either all together or per category, associated with the three different BCAAs

**Fig. 3:**
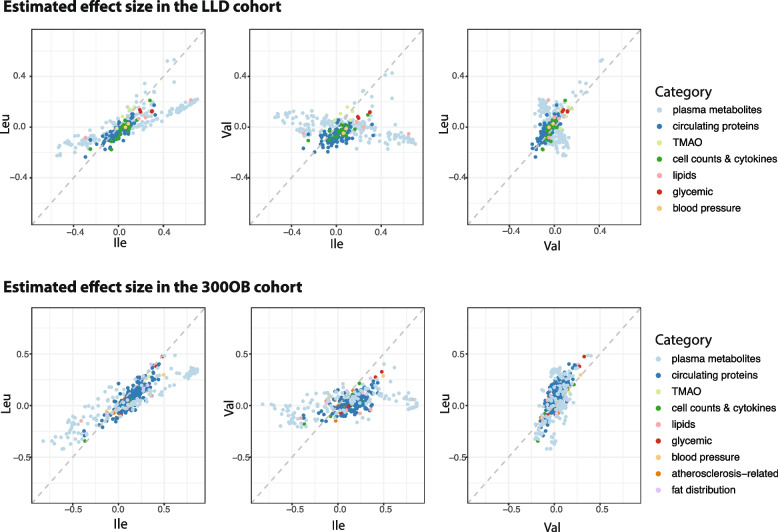
Pair-wise effect size comparison between the different BCAAs. The upper panel shows the comparisons of effect sizes in the LLD cohort and the lower panel shows the comparison in the 300OB cohort. The *x*-axis and *y*-axis represent the estimated effect sizes for isoleucine, leucine, and valine, respectively. Each dot represents an association between a BCAA and a CMD parameter, colored different based on CMD categories

Different BCAAs showed different numbers of associations with CMD parameters, and the estimated association strength also differed. Isoleucine had the highest number of associations (373 associations), followed by leucine (263) and valine (202) (Fig. 2). We found 153 CMD parameters that were associated with all three BCAAs. However, some associations were significant only for specific BCAAs: 108 parameters were only associated with isoleucine, 1 with leucine, and 24 with valine. In addition, on average, association effect sizes were largest for isoleucine (mean beta_LLD_ = 0.22), followed by leucine (mean beta_LLD_ = 0.09) and valine (mean beta_LLD_ = 0.07) (one-way ANOVA *P* = 1.71 × 10^−16^). This observation implies that different BCAAs may have a divergent impact on CMD. While zooming in on different categories of CMD parameters, we found shared associations of BCAAs for all five glycemic traits, including fasting plasma levels of glucose, insulin, HbA1c, HOMA-IR index, and diabetes status. However, BCAA-specific associations were found for lipidomic traits and proteomics (Fig. 2). The estimated association strength with NMR-based lipidomic traits was particularly strong for isoleucine when compared to the estimations with valine (Fig. 3). For example, the estimated effect size of the association between isoleucine and plasma levels of saturated fatty acids in the LLD cohort was 0.47 (*p* = 1.07 × 10^−55^), while the estimated effect size was 0.10 (*p* = 8.16 × 10^−4^) for leucine and -0.07 (*p* = 7.94 × 10^−3^) for valine (Additional file 1: Table S3). In line with this, isoleucine also showed more or stronger associations with fat distribution in the 300OB cohort. For example, plasma levels of isoleucine were significantly associated with the ratio of visceral adipose tissue to subcutaneous adipose tissue (VAT_SAT_ratio) (*β*_ile_ = 0.31), while the association was much weaker for leucine (*β*_leu_ = 0.20) and not significant for valine (*β*_val_ = 0.11) (Additional file 2: Figure S2). Moreover, only isoleucine was significantly associated with the number of carotid artery plaques (*β*_ile_ = 0.16, *p* = 0.01) in the 300OB cohort (Additional file 1: Table S3). For circulating proteins, proteins from the inflammatory panel measured in the 300OB cohort had more isoleucine-associated proteins, while CVD-related proteins measured in the LLD cohort showed more associations with valine (Additional file 1: Table S2). We did not detect many associations with cytokines (except for adiponectin, leptin, and IL-18BP) and cell counts (except for lymphocytes and leucocytes) (Additional file 2: Figure S3). In recent years, the gut microbiome has been implicated in CMD, especially through gut microbial metabolites such as TMAO, and we found both TMAO and its precursors to be associated with BCAAs (Additional file 2: Figure S4).

### Associations of valine with CMD phenotypes are dependent on obesity

**Fig. 4:**
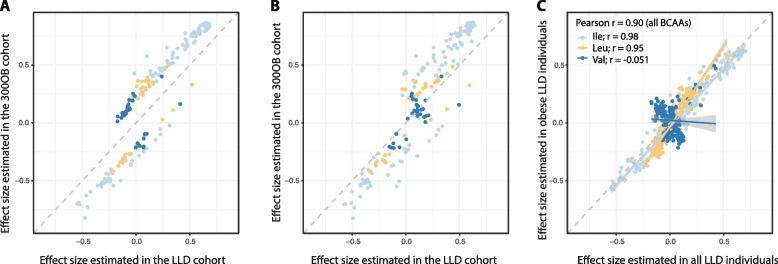
Pair-wise effect size comparison between different cohorts. **A** Comparison of effect sizes for the 202 heterogeneous associations between the LLD cohort and the 300OB cohorts. *X*-axis is the estimated effect size in the LLD cohort. *Y*-axis is the estimated effect size in the 300OB cohort. Each dot represents a heterogeneous association, colored by association with the different BCAAs: light blue for isoleucine, orange for leucine, and dark blue for valine. **B** Comparison of effect sizes for the 202 heterogeneous associations between age and BMI-matched 185 LLD individuals and the 300OB cohorts. *X*-axis represents the estimated effect size in the matched LLD obesity individuals. *Y*-axis represents the estimated effect size in the 300OB cohort. Each dot represents a heterogeneous association, colored by association with different BCAAs: light blue for isoleucine, orange for leucine, and dark blue for valine. **C** Effect size comparison between the whole LLD cohort and the subset of LLD individuals with age and BMI matched to the 300OB cohort. *X*-axis is the estimated effect size (beta value) in the whole LLD cohort. *Y*-axis is the estimated effect size (beta value) with LLD obesity individuals. Each dot represents an association between a BCAA and a CMD parameter. Dots are colored light blue for isoleucine, dark blue for valine, and orange for leucine and fitted with separate lines for the different BCAAs. The consistency between two estimations was assessed using Pearson correlation for all BCAAs together or for the different type of BCAAs separately

Out of 756 pair-wise associations for 252 CMD parameters that were tested in both the LLD and 300OB cohorts, 202 associations showed significant heterogeneity between the two cohorts (*I*^2^ > 0.75 and heterogeneity FDR < 0.05, Additional file 1: Table S5, Fig. 4), even though we had corrected for age, sex, and BMI as covariates. This observation was not due to the differences in the number of diabetes patients and glycemic traits, as the estimated effect sizes were comparable after removing T2D patients in 300OB (Pearson *r* = 0.97) (Additional file 1: Table S6, Additional file 2: Figure S4) or correcting for HOMA-IR index in the LLD cohort (Pearson *r* = 0.98) (Additional file 1: Table S7, Additional file 2: Figure S5). Interestingly, the most heterogeneous associations were related to isoleucine and lipidomic traits, and they seemed to show consistently larger effect sizes in the 300OB cohort than in the LLD cohort (Fig. 4A). Moreover, many heterogeneous associations observed for valine showed opposing directions in LLD and 300OB (Fig. 4A). This observation inspired us to hypothesize that an individual’s obesity status may explain these heterogeneous associations, which cannot be simply corrected for using BMI as a covariate. To examine this, we selected a subset of 185 individuals from the LLD cohort with matched age and BMI to the 300OB cohort. The heterogeneity in effect size in this LLD subset was not much different for leucine and isoleucine (Fig. 4B, C). Strikingly, the association directions of valine were altered in the individuals with obesity from LLD, which became consistent with the estimations in the 300OB cohort (Fig. 4C, Additional file 1: Table S8).

### Inferred causality: going beyond associations using MR

Although we identified numerous associations between BCAAs and cardiometabolic traits, and specifically with plasma lipids, using both clinical measures and detailed NMR plasma lipid levels measurements, the associations alone are insufficient to provide insights into the underlying causality. To investigate the causality of the identified associations, we performed bi-directional two-sample MR analysis using genetic variants as IVs (Fig. 1B, Additional file 3: STROBE-MR checklist) [60]. Genetic variants affecting BCAA concentrations were taken from the largest publicly available GWAS database of metabolites from the UK Biobank cohort [53, 54]. For each CMD parameter, we searched the MRC IEU Open GWAS database for summary statistics, preferably from population-based European GWAS studies to ensure similarity of SNP associations with both exposure and outcome. This resulted in data for 28 phenotypes and 217 lipoproteins (characteristics of the included GWASs can be found in Additional file 1: Table S9). As some of the available studies were based on UK Biobank data (see Additional file 1: Table S9), there was a sample overlap between exposure and outcome data, and some of the MR analyses may not be a truly two-sample MR.

**Fig. 5:**
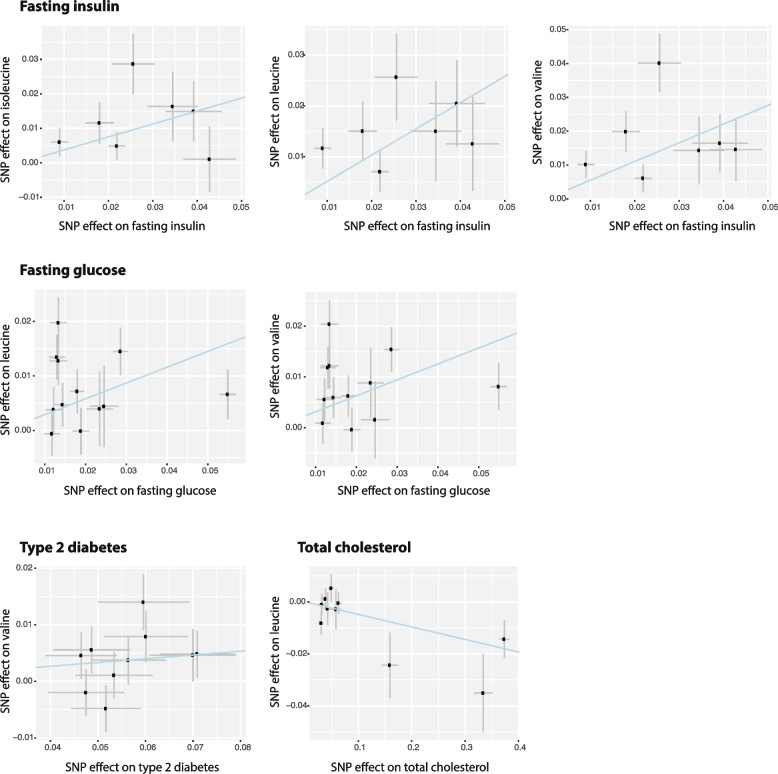
Mendelian randomization analysis. *X*-axes show the SNP–exposure effect. *Y*-axes show the SNP–outcome effect (SEs denoted as segments). The plots show the significant causal estimates of fasting insulin, fasting glucose, type 2 diabetes, and total cholesterol on BCAAs. The blue line corresponds to the causal estimate obtained using the inverse variance weighted method

Obesity has previously been reported to causally contribute to both CMD parameters and BCAAs [3, 4, 41]. Therefore, we performed two types of MR analyses to deal with the confounding effect of BMI and removed causal estimates that failed sensitivity analyses (see the “Methods” section, Additional file 1: Tables S10-S11). In the MVMR analysis, including BMI as an additional exposure covariate revealed eight potential causal links from three phenotypes to BCAAs: increases in triglyceride levels and frequency of T2D and a decrease in HDL cholesterol levels were all associated with an increase in BCAA levels (Additional file 1: Table S10); however, the heterogeneity of these associations was high. A UVMR analysis, performed after removing BMI-associated SNPs, identified seven significant results linking four traits to BCAA levels. For example, for each 1 SD increase in genetically predicted leucine, we saw a 0.52 SD increase in fasting insulin, a 0.29 SD increase in fasting glucose and a 0.05 SD decrease in total cholesterol levels (Additional file 1: Table S11, Fig. 5). As BCAA levels are highly correlated, we used the combined levels of all three BCAAs as the exposure when estimating the causal effect of BCAAs on CMD parameters. However, neither method could detect any significant causal relationships from BCAAs to phenotypes in our dataset as these MR results failed QC sensitivity analyses (Additional file 1: Table S11). Altogether, our results suggest altered plasma BCAA levels are more likely the outcome of metabolic syndromes. This was further confirmed by our observation of a large number of causal effects of lipidomic traits on BCAAs: 504 significant estimates for 185 unique lipidomic traits using MVMR (Additional file 1: Table S10) and 252 significant estimates for 96 traits using UVMR (Additional file 1: Table S10). All reported UVMR effects passed the pleiotropy and heterogeneity checks performed using the MR Egger and MR PRESSO methods. However, a high degree of heterogeneity for most of the MR effects was observed based on Cochran's *Q*-test. This implies complexity in causality and suggests some caution in data interpretation.

## Discussion

Valine, leucine, and isoleucine—the BCAAs—are essential amino acids that are used for protein synthesis but also play important roles in signaling pathways. Increased plasma BCAA concentrations have been found to increase the risk of IR and diabetes and some causal pathways have been proposed [3, 12, 14–16], but their relation with other CMD-related parameters is not completely understood. Previous studies investigated the associations of BCAAs with CMD using only a limited number of CMD phenotypes, or in sex-, age- or disease-specific cohorts with small sample sizes, making it difficult to generalize their findings [61–63]. Here, we made use of very detailed clinical and omics-guided phenotyping data available for 1694 individuals from two cohorts, a general population cohort and an overweight/obese cohort of older individuals with high cardiovascular risk, to uniformly profile BCAA relations with common CMD-related parameters. We systematically explored the relationships between BCAA concentrations and a large panel of 537 CMD parameters and provided an estimate of causality of these associations using MR.

We created an inventory of 827 significant BCAA associations with CMD-related parameters. We not only replicated previous findings of associations with glycemic-related factors and all three BCAAs [4, 41], we also detected a large number of associations for a wide range of CMD-related parameters, particularly with lipoproteins and circulating protein levels. We identified fewer BCAA associations with cytokines, cell counts and TMAO metabolites.

BCAAs appear to have both common and specific associations. A large set of available CMD parameters allowed us to accurately compare the association effects between isoleucine, leucine, and valine. Under the presumption that these three BCAAs have similar functions, many studies have missed the specific effects of each BCAA and broaden their conclusions based on the study of single amino acids and/or of their individual effects to all BCAAs. It is becoming increasingly clear that the associations of specific BCAAs with CVD may differ substantially [64, 65]. The largest currently recognized difference is for leucine, which is known to have slightly different cellular functions compared to valine and isoleucine [66, 67]. Based on our findings, it appears that the associations with glycemic traits are shared across all three BCAAs; however, for other CMD parameters, we found a large number of specific associations, especially the associations between isoleucine and circulating lipoprotein and protein levels. These findings are consistent with a recent study proposing that both isoleucine and valine were responsible for the adverse metabolic effects of BCAAs in mice, with a particularly clear effect for isoleucine [65]. Furthermore, in our study, only isoleucine was associated with the number of carotid artery plaques, suggesting that it would be prudent to specify BCAAs when evaluating the CMD etiology. Based on the associations demonstrated in the current study, it would be plausible to consider isoleucine in clinical and experimental research on atherosclerotic plaques.

Another important finding is the relationship of BCAAs, obesity, and lipid metabolism. As noted above, BCAAs appear to have particularly strong associations with lipid profile. We found that these associations depend on age and BMI. First, effect sizes were higher in the 300OB cohort of overweight/obese individuals, who were also considerably older. Second, valine showed a large number of associations with opposing directions across the two cohorts, a conflict that was resolved after matching for age and BMI of individuals from the two cohorts.

While BCAAs clearly play a role in IR and diabetes, previous studies reported conflicting results on the direction of causal relationship in these associations: Lotta et al. reported that changes in BCAA levels contribute to IR and the incidence of type 2 diabetes [45], whereas more recent studies showed evidence of a BCAA effect on IR [46, 47]. Our results support the potential causal effect of fasting insulin and glucose on BCAA levels. In addition, we see multiple lipid-related causal links, e.g., total cholesterol levels were potentially causally related to a decrease in BCAA levels. Additionally, we observed that many NMR-based lipoproteins showed a causal effect on BCAA levels. However, these results should be interpreted with caution because of a strong correlation between the lipid traits and a high heterogeneity of the resulting MR estimates. No significant causal links from BCAA levels to CMD-related parameters were detected, potentially due to the lack of strong genetic instruments for this analysis, which may indicate that altered plasma BCAA levels are more likely to be the outcome of the metabolic syndrome. Further studies in other datasets, including non-European populations, are required to estimate the generalizability of the study results.

We acknowledge several limitations. This cohort-based, epidemiological study primarily provided population-level association evidence, as well as future directions for investigating the role of BCAAs in CMD. However, the cross-sectional association design of the current study led to several limitations. First, the identified associations may not directly reflect the observations from animal-based, mechanistic biology studies, as experimental settings vary greatly. Second, our association approach assumes linear relationships between BCAAs and CMD parameters, which might not always be the case. Larger studies using non-linear models are needed to capture these complex relationships. Third, the identified associations do not imply causality. While we did employ MR to estimate directions of causality for these associations, only a few causal relationships were supported, and these were predominantly in the direction from CMD parameters to BCAA levels. However, the causal effects of BCAAs on CMD parameters were more difficult to estimate due to the lower number of BCAA-associated SNPs available for the analyses. Lastly, violation of assumptions for the MR analyses may occur even when performing a rigorous sensitivity analysis, potentially leading to false conclusions. Further studies are needed to elucidate the potential underlying mechanisms of the identified associations and causal links.

## Conclusions

We performed a cohort-based association study of BCAAs with cardiometabolic parameters. Our findings can serve as an extensive catalog to propagate future BCAA and CMD research. Interestingly, BCAAs appear to be a heterogeneous group of amino acids with potentially diverse CMD-associated effects, which suggests they should be targeted separately in biomedical, clinical and experimental research.

## Supplementary information

**Additional file 1:** **Tables S1-S10.** **Table S1** - Phenotype description. **Table S2** - Association of BCAAs with potential covariates. **Table S3** - The results of association analysis between BCAAs and CMD parameters adjusted for age, sex and BMI. **Table S4** - The results of association analysis between BCAAs and CMD parameters adjusted for age, sex, BMI and eGFR. **Table S5** - Meta analysis of association results in LLD and 300OB cohorts. **Table S6** - The results of association analysis between BCAAs and NMR lipoproteins performed in the 300OB cohort adjusted for age, sex and BMI after removing individuals with DM. **Table S7** - The results of association analysis between BCAAs and NMR lipoproteins in LLD cohort adjusted for age, sex, BMI and HOMA-IR. **Table S8** - The results of association analysis between BCAAs and NMR lipoproteins in a subset of the LLD cohort, that was matched by age and BMI with the 300OB cohort. **Table S9** - The list of CMD parameters used in MR analyses and their accession ids. **Table S10** - The results of multivariable MR analyses. **Table S11** - The results of univariable MR analyses

**Additional file 2:** **Figures S1-S5.** **Figure S1** - Effect size comparison with and without adjustment for eGFR. **Figure S2** -Effect size comparisons of BCAAs association with fat distribution in the 300OB cohort. **Figure S3** - Effect size comparisons of BCAA associations with cytokines and cell counts in both the LLD and 300OB cohorts. **Figure S4** - Effect size comparisons of BCAA associations with TMAO and its precursors in both the LLD and 300OB cohorts. **Figure S5** - Effect size comparison with and without correcting for diabetes and insulin resistance

**Additional file 3.** STROBE-MR checklist. STROBE-MR checklist - A checklist of recommended items to address in reports of Mendelian randomization studies

## Data Availability

All summary statistics and association results are included in this published article and its supplementary information files. Due to informed consent regulation and the sensitive nature of clinical data, detailed datasets of individual participants of the LifeLines DEEP and 300OB cohorts can only be made available upon request to the LifeLines organization and the Human Functional Genomics Project, respectively. This includes the submission of a letter of intention to the corresponding data access committee [the LifeLines Data Access Committee for the LifeLines DEEP data (research@lifelines.nl) and the Human Functional Genomics Data Access Committee for 300OB data (Martin Jaeger, e-mail: Martin.Jaeger@radboudumc.nl)]. Datasets can be made available under a data transfer agreement, and the data usage access is subject to local rules and regulations.

## Abbreviations

BCAAs: Branched-chain amino acids
CMD: Cardiometabolic diseases
T2D: Type 2 diabetes
IR: Insulin resistance
mTOR: Mammalian target of rapamycin
CVD: Cardiovascular diseases
TMAO: Trimethylamine N-oxide
LLD: LifeLines DEEP
BMI: Body mass index
NMR: Nuclear magnetic resonance
IMT: Intima-media thickness
