# Supplementary material for: Systematic analysis of relationships between plasma branched-chain amino acid concentrations and cardiometabolic parameters: an association and Mendelian randomization study
Source: BMC Med. 2022 Dec 15;20:485. doi: 10.1186/s12916-022-02688-4 (PMC9753387; doi:10.1186/s12916-022-02688-4)

Additional file 2

Systematic analysis of relationships between plasma branched-chain amino acid concentrations and cardiometabolic parameters: an association and Mendelian randomization study

Table of Contents

Figure S1: Effect size comparison with and without adjustment for eGFR. 2

Figure S2: Effect size comparisons of BCAAs association with fat distribution in the 300OB cohort. 3

Figure S3: Effect size comparisons of BCAA associations with cytokines and cell counts in both the LLD and 300OB cohorts. 4

Figure S4: Effect size comparisons of BCAA associations with TMAO and its precursors in both the LLD and 300OB cohorts. 5

Figure S5: Effect size comparison with and without correcting for diabetes and insulin resistance. 6

Figure S1: Effect size comparison with and without adjustment for eGFR.

X-axis refers to the estimated effect size (beta value) without adjustment for eGFR. Y-axis refers to the estimated effect size (beta value) after adjustment for eGFR. Each dot represents an association between a BCAA and a CMD parameter. The dots are colored – light blue for isoleucine, dark blue for valine and orange for leucine – with fitted lines drawn separately for different BCAAs. The consistency between two estimations was assessed using Pearson correlation.

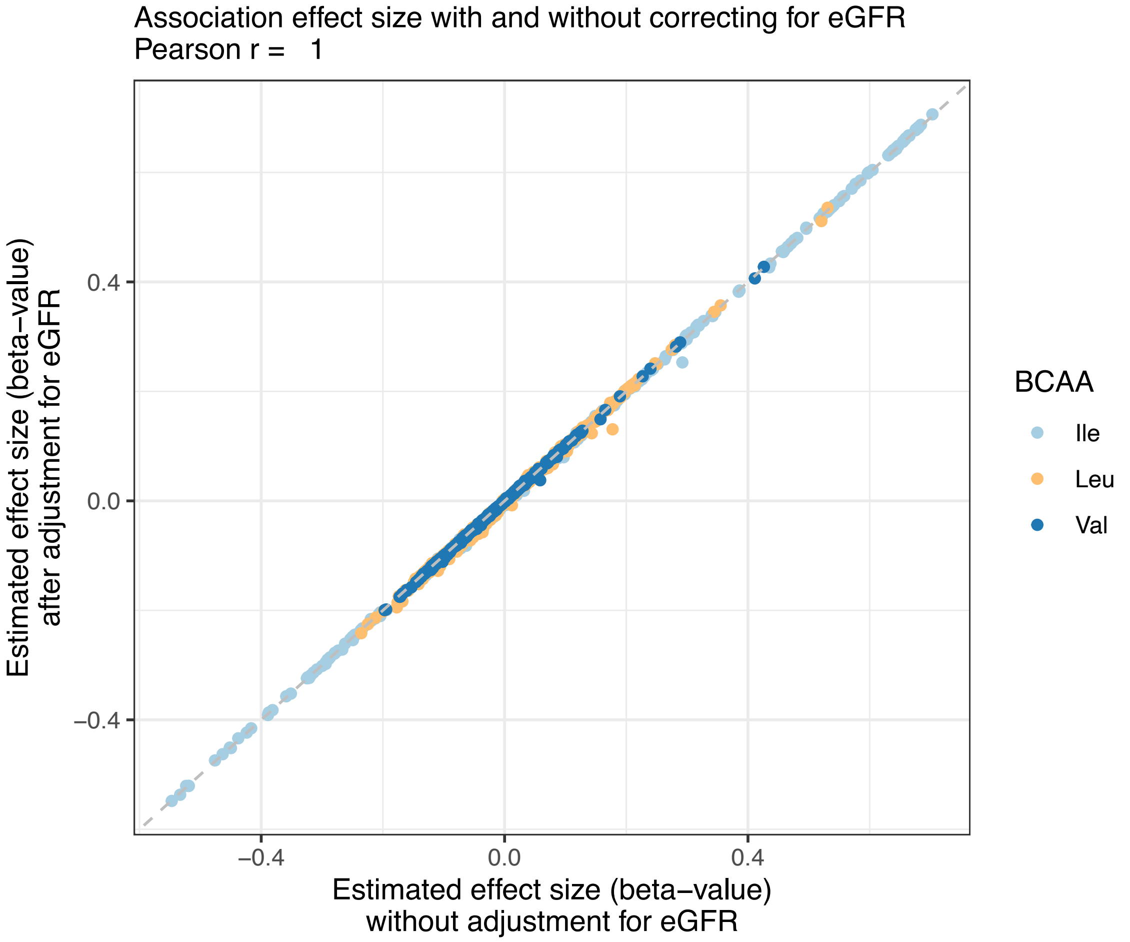

Figure S2: Effect size comparisons of BCAAs association with fat distribution in the 300OB cohort.

The estimated effect sizes are shown as forest plots for the different BCAAs separately. The red dots with bar represent the estimated effect sizes with 95% confidence intervals in the 300OB cohort. If the association is significant at FDR < 0.05 level, the effect is presented as a filled dot. Otherwise, the effect is presented as an open dot.

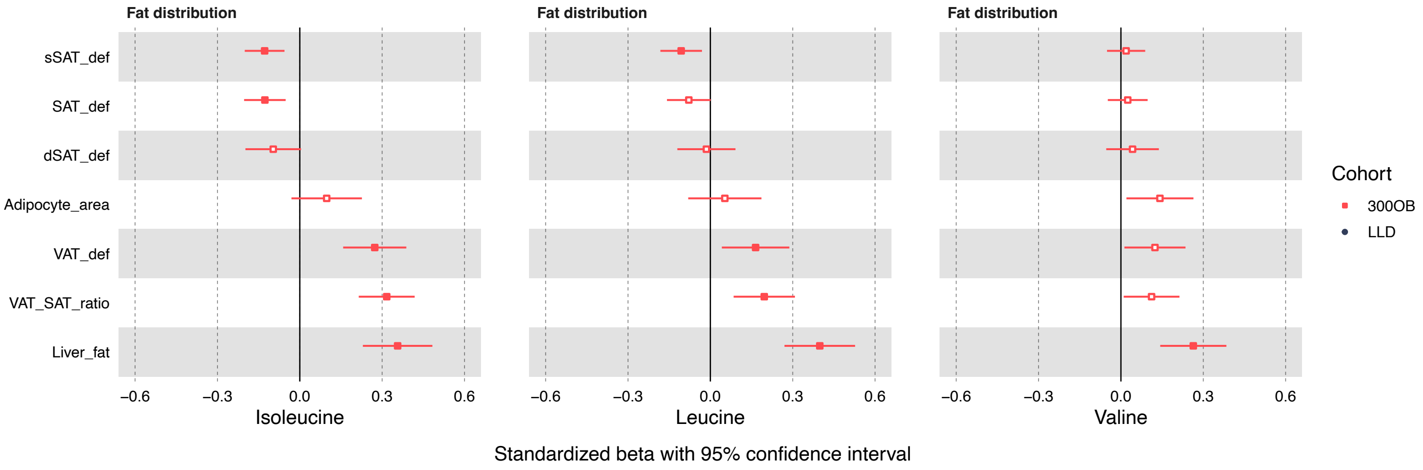

Figure S3: Effect size comparisons of BCAA associations with cytokines and cell counts in both the LLD and 300OB cohorts.

The estimated effect sizes are shown as forest plots for the different BCAAs separately. The red dots with bar represent to the estimated effect sizes with 95% confidence intervals in the 300OB cohort. The black dots with bar represent to the estimated effect sizes with 95% confidence intervals in the LLD cohort. If the association was significant at FDR < 0.05 level, the effect is presented as a filled dot. Otherwise, the effect is presented as an open dot.

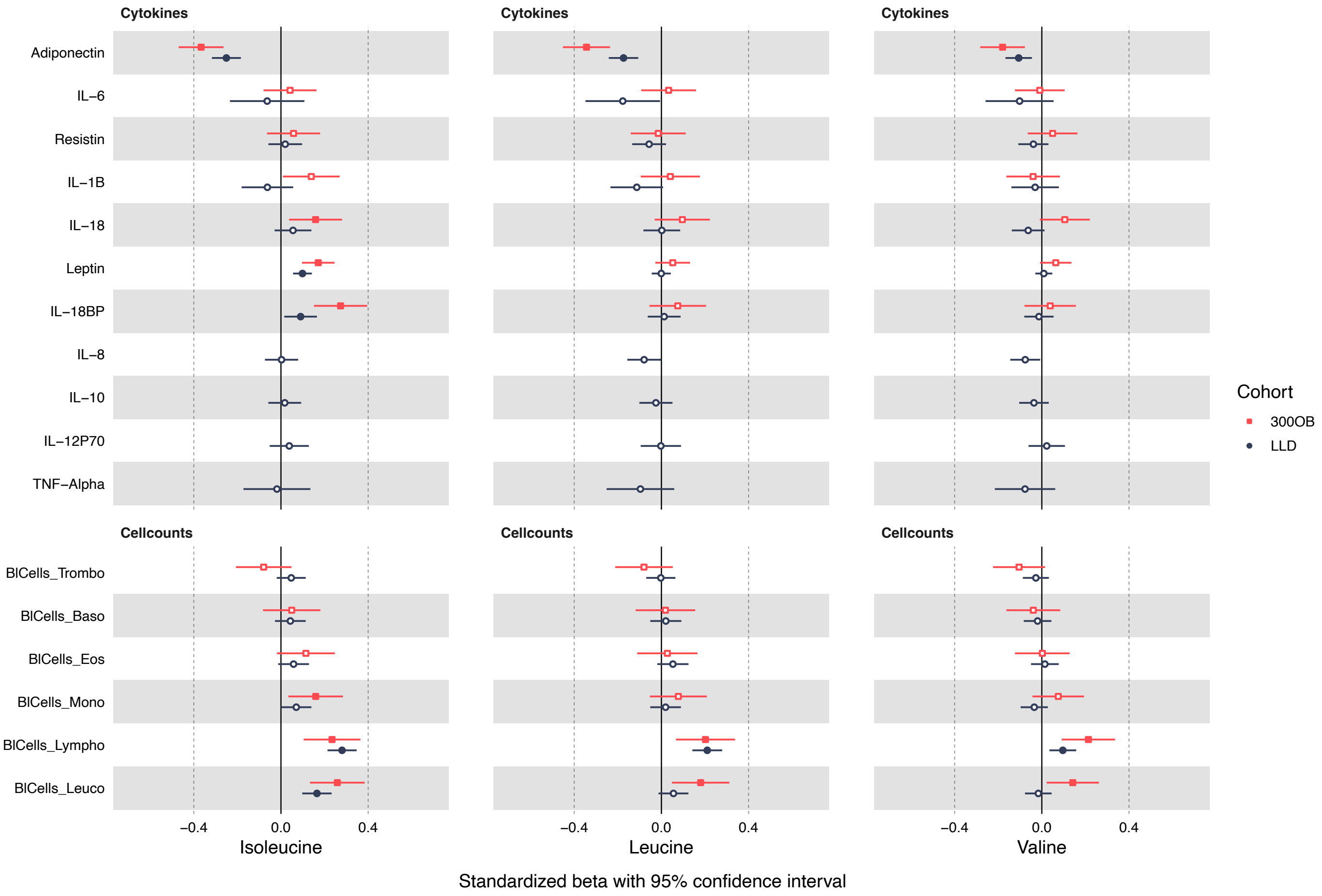

Figure S4: Effect size comparisons of BCAA associations with TMAO and its precursors in both the LLD and 300OB cohorts.

The estimated effect sizes are shown as forest plots for the different BCAAs separately. The red dots with bar represent to the estimated effect sizes with 95% confidence intervals in the 300OB cohort. The black dots with bar represent to the estimated effect sizes with 95% confidence intervals in the LLD cohort. If the association was significant at FDR < 0.05 level, the effect is presented as a filled dot. Otherwise, the effect is presented as an open dot.

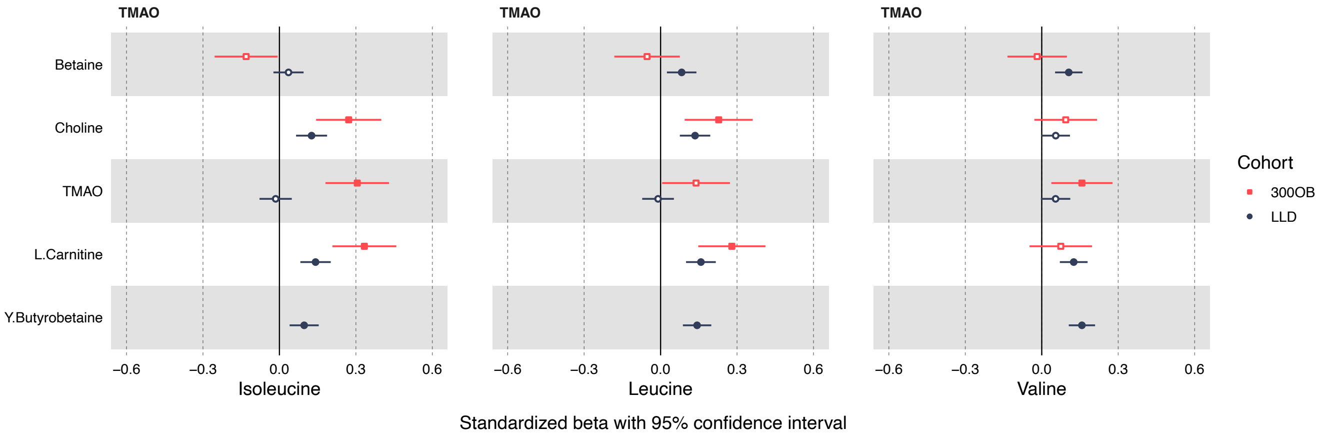

Figure S5: Effect size comparison with and without correcting for diabetes and insulin resistance.

A) The effect size comparison with and without T2D patients in the 300OB cohort. **B**) The effect size comparison with and without adjustment for HOMA-IR in the LLD cohort. X-axis refers to the estimated effect size (beta value) without adjustment. Y-axis refers to the estimated effect size (beta value) after adjustment. Each dot represents an association between a BCAA and a CMD parameter. The dots are colored light blue for isoleucine, dark blue for valine and orange for leucine, with fitted lines drawn separately for the different BCAAs.

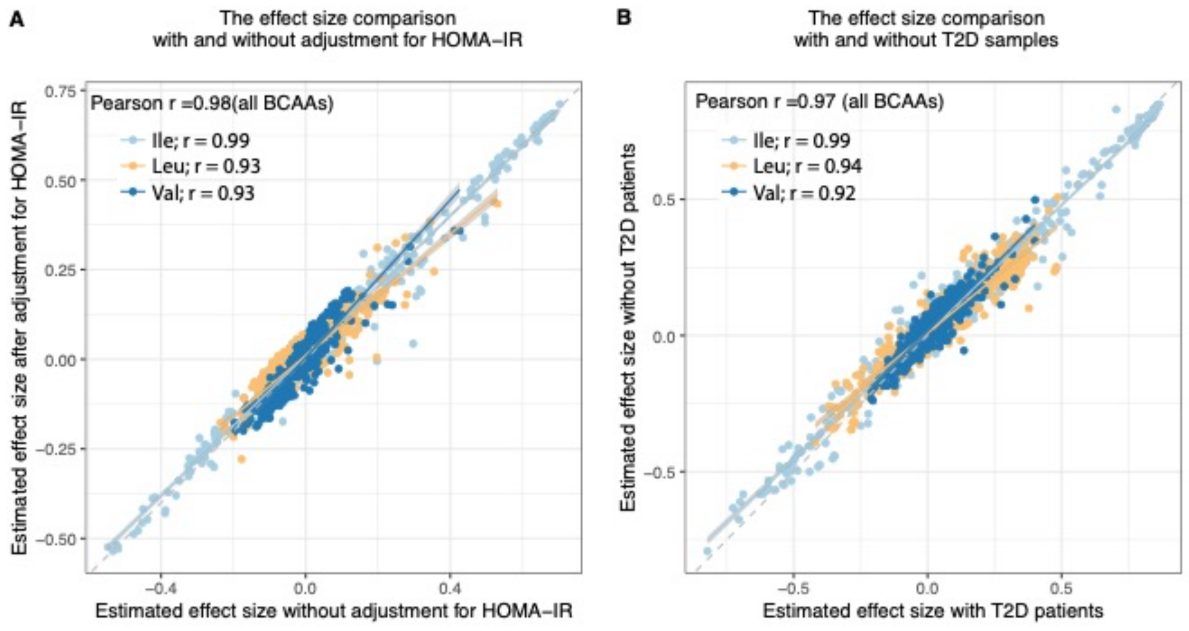

Supplement: Supplementary file 2 — Additional file 2: Figures S1-S5. Figure S1 - Effect size comparison with and without adjustment for eGFR. Figure S2 -Effect size comparisons of BCAAs association with fat distribution in the 300OB cohort. Figure S3 - Effect size comparisons of BCAA associations with cytokines and cell counts in both the LLD and 300OB cohorts. Figure S4 - Effect size comparisons of BCAA associations with TMAO and its precursors in both the LLD and 300OB cohorts. Figure S5 - Effect size comparison with and without correcting for diabetes and insulin resistance [file 12916_2022_2688_MOESM2_ESM.pdf]
