# Supplementary material for: Systematic analysis of relationships between plasma branched-chain amino acid concentrations and cardiometabolic parameters: an association and Mendelian randomization study
Source: BMC Med. 2022 Dec 15;20:485. doi: 10.1186/s12916-022-02688-4 (PMC9753387; doi:10.1186/s12916-022-02688-4)
Supplement: Supplementary file 3 — Additional file 3. STROBE-MR checklist. STROBE-MR checklist - A checklist of recommended items to address in reports of Mendelian randomization studies [file 12916_2022_2688_MOESM3_ESM.pdf]

Additional file 3:

STROBE-MR checklist

of recommended items to address in reports of Mendelian randomization studies[1 2]

Item No.	Section	Checklist item	Page No.	Relevant text from manuscript
1	**TITLE and ABSTRACT**	Indicate Mendelian randomization (MR) as the study's design in the title and/or the abstract if that is a main purpose of the study	1	Systematic analysis of relationships between plasma branched-chain amino acid concentrations and cardiometabolic parameters: an association and Mendelian randomization study
	**INTRODUCTION**			
2	**Background**	Explain the scientific background and rationale for the reported study. What is the exposure? Is a potential causal relationship between exposure and outcome plausible? Justify why MR is a helpful method to address the study question	6	Taken together, the relationship between BCAAs and some components of CMD, such as IR/diabetes, have been extensively studied, but the relationship of BCAAs and other CMD parameters has yet to be established, as most studies to date are either pre-clinical or performed for sex- and disease-specific conditions. In addition, while many of the mechanistic studies in animal models have provided evidence of individual-level causality, systematic evaluation in human cohorts is crucial to provide population-level evidence and identify the potential etiological roles of BCAAs in CMD. Moreover, previous results about the direction of the causal relationship between BCAAs and CMD are conflicting. For example, a causal role for BCAAs in IR was supported in a study by Lotta et al [45], while reverse causality of IR on BCAAs has been suggested by other studies [46, 47].
3	**Objectives**	State specific objectives clearly, including pre-specified causal hypotheses (if any). State that MR is a method that, under specific assumptions, intends to estimate causal effects	6	Here, we present a systematic, cross-sectional association analysis between fasting plasma concentrations of BCAAs and a large panel of 537 parameters (including clinical CMD measures such as non-invasive measures of atherosclerosis, fat distribution, circulating CVD-related proteins, plasma metabolites and inflammatory cell counts and immune cytokines) in 1,405 individuals from the general population–based LifeLines DEEP (LLD) cohort [48] and 294 overweight/obese individuals from 300OB the cohort [49]. In this study, we (1) establish association relationships between BCAAs and CMD-related traits that are independent of age, sex, BMI and other potential covariates, (2) estimate and compare the association strength between different BCAAs and (3) interrogate the potential causal direction of associations using a bi-directional Mendelian randomization (MR) approach.
	**METHODS**			

4	**Study design and data sources**		Present key elements of the study design early in the article. Consider including a table listing sources of data for all phases of the study. For each data source contributing to the analysis, describe the following:		
		a)	Setting: Describe the study design and the underlying population, if possible. Describe the setting, locations, and relevant dates, including periods of recruitment, exposure, follow-up, and data collection, when available.	13	Summary statistics for all of the cardiometabolic traits were obtained from the OpenGWAS database, where we aimed recover data from the GWAS with the highest sample size, preferably based on European samples, for each CMD parameter tested in the association analysis (see **Additional file 1: Table S9** for the list of studies and the trait description).
		b)	Participants: Give the eligibility criteria, and the sources and methods of selection of participants. Report the sample size, and whether any power or sample size calculations were carried out prior to the main analysis		see above
		c)	Describe measurement, quality control and selection of genetic variants	12-13	The genetic variants used as instrumental variables for MR analyses and their effect sizes were obtained from publicly available summary statistics for genome-wide association studies (GWASs) on BCAAs and cardiometabolic traits. BCAA-associated SNPs were taken from the largest GWAS on metabolites to date performed in the UK Biobank and available in the OpenGWAS database [54] under accession IDs met-d-Val, met-d-Ile and met-d-Leu. Summary statistics for all of the cardiometabolic traits were obtained from the OpenGWAS database, where we aimed recover data from the GWAS with the highest sample size, preferably based on European samples, for each CMD parameter tested in the association analysis (see **Additional file 1: Table S9** for the list of studies and the trait description). Genetic variants were clumped using $r^2 < 0.001$ in 1000G EUR samples. Proxies were added automatically by the TwoSampleMR R package.
		d)	For each exposure, outcome, and other relevant variables, describe methods of assessment and diagnostic criteria for diseases		NA
		e)	Provide details of ethics committee approval and participant informed consent, if relevant		NA
5	**Assumptions**		Explicitly state the three core IV assumptions for the main analysis (relevance, independence and exclusion restriction) as well assumptions for any additional or sensitivity analysis	13	Instrumental variables (IVs) used in MR need to fulfill three major assumptions: (1) the IVs should be associated with the exposure, (2) the IVs should not share a common cause with the outcome and (3) the IVs should affect the outcome only through the exposure.

6	**Statistical methods: main analysis**		Describe statistical methods and statistics used		
		a)	Describe how quantitative variables were handled in the analyses (i.e., scale, units, model)	13	see **Additional file 1: Table S9** for the list of studies and the trait description
		b)	Describe how genetic variants were handled in the analyses and, if applicable, how their weights were selected	13	Summary statistics for all of the cardiometabolic traits were obtained from the OpenGWAS database
		c)	Describe the MR estimator (e.g. two-stage least squares, Wald ratio) and related statistics. Detail the included covariates and, in case of two-sample MR, whether the same covariate set was used for adjustment in the two samples	13	To calculate univariable MR (UVMR) estimates, we used Wald ratios meta-analyzed by the inverse variance weighted (IVW) method.
		d)	Explain how missing data were addressed		NA
		e)	If applicable, indicate how multiple testing was addressed	14	We performed the analyses in both directions and corrected for multiple testing using the Benjamini-Hochberg method separately for each group of phenotypes (general cardiometabolic traits and NMR lipoproteins) and for each direction.
7	**Assessment of assumptions**		Describe any methods or prior knowledge used to assess the assumptions or justify their validity	14	BMI has previously been shown to affect both BCAA levels and CMD parameters. Moreover, BMI, BCAAs and CMD parameters have shared associated genetic variants. To overcome the potential violation of the 3rd MR assumption in the UVMR analyses, we removed BMI-associated SNPs published by the GIANT consortium [58] from the list of genetic variants. In addition, we corrected for the effect of BMI using multivariable MR analyses (MVMR).
8	**Sensitivity analyses and additional analyses**		Describe any sensitivity analyses or additional analyses performed (e.g. comparison of effect estimates from different approaches, independent replication, bias analytic techniques, validation of instruments, simulations)	13-14	To reduce the chances of violating these assumptions, extensive QC and sensitivity analyses were performed on the candidate MR results. In detail, results were only considered when they met the following criteria: (1) MR results were based on three or more SNPs, as this allowed us to perform the sensitivity analyses listed below, (2) MR results showed a Benjamini-Hochberg-corrected *p* value < 0.05 using IVW and nominally significant results (*p* value < 0.05) using two other MR approaches (weighted median and MR PRESSO test [56, 57]), (3) MR results did not show indications of horizontal pleiotropy or heterogeneity, as estimated using MR Egger [57] (intercept *p* value > 0.05) and the MR PRESSO [56] outlier-adjusted test (*p* value < 0.05) that estimates the pleiotropy and tries to correct for it by removing outliers, (4) MR results were not driven by single SNPs, as tested using leave-one-out analyses (no SNP after exclusion resulting in IVW MR *p* value > 0.05), or (5) genetic instruments were strong as estimated using *F*-statistics (*F* > 10). We also estimated heterogeneity using Cochran's *Q*-test but did not filter out the results based on this measure.

					Sensitivity analyses of the MVMR results were performed by the MVMR v. 0.3 R package [59], which estimates the strength of the genetic instruments (*F*-statistics, which we required to be > 10) and heterogeneity using Cochran's *Q*-test.
9	**Software and pre-registration**				
		a)	Name statistical software and package(s), including version and settings used	12-14	To determine causality between BCAAs and associated factors, two-sample bi-directional MR was performed using the R package TwoSampleMR v.0.5.6 with default settings. Sensitivity analyses of the MVMR results were performed by the MVMR v. 0.3 R package
		b)	State whether the study protocol and details were pre-registered (as well as when and where)		NA
	**RESULTS**				
10	**Descriptive data**				
		a)	Report the numbers of individuals at each stage of included studies and reasons for exclusion. Consider use of a flow diagram	17	characteristics of the included GWASs can be found in **Additional file 1: Table S9**
		b)	Report summary statistics for phenotypic exposure(s), outcome(s), and other relevant variables (e.g. means, SDs, proportions)		NA
		c)	If the data sources include meta-analyses of previous studies, provide the assessments of heterogeneity across these studies		NA
		d)	For two-sample MR:<br>i. Provide justification of the similarity of the genetic variant-exposure associations between the exposure and outcome samples<br>ii. Provide information on the number of individuals who overlap between the exposure and outcome studies	17	For each CMD parameter, we searched the MRC IEU Open GWAS database for summary statistics, preferably from population-based European GWAS studies to ensure similarity of SNP associations with both exposure and outcome. This resulted in data for 28 phenotypes and 217 lipoproteins (characteristics of the included GWASs can be found in **Additional file 1: Table S9**). As some of the available studies were based on UK Biobank data (see **Additional file 1: Table S9**), there was a sample overlap between exposure and outcome data, and some of the MR analyses may not be a truly two-sample MR.
11	**Main results**				

		a)	Report the associations between genetic variant and exposure, and between genetic variant and outcome, preferably on an interpretable scale		NA
		b)	Report MR estimates of the relationship between exposure and outcome, and the measures of uncertainty from the MR analysis, on an interpretable scale, such as odds ratio or relative risk per SD difference	18	For example, for each 1 SD increase in genetically predicted leucine, we saw a 0.52 SD increase in fasting insulin, a 0.29 SD increase in fasting glucose and a 0.05 SD decrease in total cholesterol levels (**Additional file 1: Table S11**, **Figure 5**).
		c)	If relevant, consider translating estimates of relative risk into absolute risk for a meaningful time period		NA
		d)	Consider plots to visualize results (e.g. forest plot, scatterplot of associations between genetic variants and outcome versus between genetic variants and exposure)		Figure 5
12	**Assessment of assumptions**				
		a)	Report the assessment of the validity of the assumptions	17	Therefore, we performed two types of MR analyses to deal with the confounding effect of BMI and removed causal estimates that failed sensitivity analyses (see Methods, **Additional file 1: Tables S10-S11**).
		b)	Report any additional statistics (e.g., assessments of heterogeneity across genetic variants, such as I^2, Q statistic or E-value)	18	However, a high degree of heterogeneity for most of the MR effects was observed based on Cochran's *Q*-test.
13	**Sensitivity analyses and additional analyses**				
		a)	Report any sensitivity analyses to assess the robustness of the main results to violations of the assumptions	17	Therefore, we performed two types of MR analyses to deal with the confounding effect of BMI and removed causal estimates that failed sensitivity analyses (see Methods, **Additional file 1: Tables S10-S11**).
		b)	Report results from other sensitivity analyses or additional analyses		

		c)	Report any assessment of direction of causal relationship (e.g., bidirectional MR)	18	However, neither method could detect any significant causal relationships from BCAAs to phenotypes in our dataset as these MR results failed QC sensitivity analyses (**Additional file 1: Table S11**).
		d)	When relevant, report and compare with estimates from non-MR analyses		NA
		e)	Consider additional plots to visualize results (e.g., leave-one-out analyses)		NA
	**DISCUSSION**				
14	**Key results**		Summarize key results with reference to study objectives	20	Our results support the potential causal effect of fasting insulin and glucose on BCAA levels. In addition, we see multiple lipid-related causal links, e.g. total cholesterol levels were potentially causally related to a decrease in BCAA levels.
15	**Limitations**		Discuss limitations of the study, taking into account the validity of the IV assumptions, other sources of potential bias, and imprecision. Discuss both direction and magnitude of any potential bias and any efforts to address them	21	While we did employ MR to estimate directions of causality for these associations, only a few causal relationships were supported, and these were predominantly in the direction from CMD parameters to BCAA levels. However, the causal effects of BCAAs on CMD parameters were more difficult to estimate due to the lower number of BCAA-associated SNPs available for the analyses. Lastly, violation of assumptions for the MR analyses may occur even when performing a rigorous sensitivity analysis, potentially leading to false conclusions. Further studies are needed to elucidate the potential underlying mechanisms of the identified associations and causal links.
16	**Interpretation**				
		a)	Meaning: Give a cautious overall interpretation of results in the context of their limitations and in comparison with other studies	20	While BCAAs clearly play a role in IR and diabetes, previous studies reported conflicting results on the direction of causal relationship in these associations: Lotta et al. reported that changes in BCAA levels contribute to IR and the incidence of type 2 diabetes [45], whereas more recent studies showed evidence of a BCAA effect on IR [46, 47]. Our results support the potential causal effect of fasting insulin and glucose on BCAA levels. In addition, we see multiple lipid-related causal links, e.g. total cholesterol levels were potentially causally related to a decrease in BCAA levels. Additionally, we observed that many NMR-based lipoproteins showed a causal effect on BCAA levels. However, these results should be interpreted with caution because of a strong correlation between the lipid traits and a high heterogeneity of the resulting MR estimates. No significant causal links from BCAA levels to CMD-related parameters were detected, potentially due to the lack of strong genetic instruments for this analysis, which may indicate that altered plasma BCAA levels are more likely to be the outcome of the metabolic syndrome.

	b)	Mechanism: Discuss underlying biological mechanisms that could drive a potential causal relationship between the investigated exposure and the outcome, and whether the gene-environment equivalence assumption is reasonable. Use causal language carefully, clarifying that IV estimates may provide causal effects only under certain assumptions	21	Further studies are needed to elucidate the potential underlying mechanisms of the identified associations and causal links.
	c)	Clinical relevance: Discuss whether the results have clinical or public policy relevance, and to what extent they inform effect sizes of possible interventions	20	Our results support the potential causal effect of fasting insulin and glucose on BCAA levels.
17	**Generalizability**	Discuss the generalizability of the study results (a) to other populations, (b) across other exposure periods/timings, and (c) across other levels of exposure	20	Further studies in other datasets, including non-European populations, are required to estimate the generalizability of the study results.
	**OTHER INFORMATION**			
18	**Funding**	Describe sources of funding and the role of funders in the present study and, if applicable, sources of funding for the databases and original study or studies on which the present study is based	24	This project was funded by grants from the Top Institute Food and Nutrition, Wageningen, the Netherlands to C.W. (TiFN GH001); the Netherlands Organization for Scientific Research (NWO) to J.F. (NWO-VIDI 864.13.013 and NWO-VICI VI.C.202.022), A.Z. (NWO-VIDI 016.178.056) and D.V.Z. (NWO-VENI 194.006); the NWO Gravitation grant Exposome-NL (024.004.017) and CardioVasculair Onderzoek Nederland to N.R., M.G.N., A.Z., F.K. and J.F. (IN CONTROL II, CVON 2018-27). The NMR metabolomics profiling was supported by BBMRI-NL, a research infrastructure financed by NWO. A.Z. holds a Rosalind Franklin Fellowship from the University of Groningen and a European Research Council (ERC) Starting Grant (715772). J.F. holds an ERC Consolidator grant (grant agreement No. 101001678). C.W. and J.F. were supported by the Netherlands Organ-on-Chip Initiative, an NWO Gravitation project (024.003.001) funded by the Ministry of Education, Culture and Science of the government of the Netherlands. C.W. also received a Spinoza Prize from NWO (SPI 92-266) and an FP7/2007-2013/ERC Advanced Grant (agreement 2012-322698). We thank the participants and staff of the Lifelines DEEP cohort for their collaboration. The study was approved by the UMCG's review board, ref. M12.113965. We thank Jackie Dekens, Mathieu Platteel, Maria Carmen Cenit, Astrid Maatman and Jody Arends for project management and technical support and Kate Mc Intyre for editing the manuscript.
19	**Data and data sharing**	Provide the data used to perform all analyses or report where and how the data can be accessed, and reference these sources in the article. Provide the statistical code needed to reproduce	21	All summary statistics and association results are included in this published article and its supplementary information files. Due to informed consent regulation and the sensitive nature of clinical data, detailed datasets of individual participants of the Lifelines DEEP and 300OB cohorts can only be made available upon request to the

		the results in the article, or report whether the code is publicly accessible and if so, where		LifeLines organization and the Human Functional Genomics Project, respectively. This includes the submission of a letter of intention to the corresponding data access committee [the LifeLines Data Access Committee for the Lifelines DEEP data (research@lifelines.nl) and the Human Functional Genomics Data Access Committee for 300OB data (Martin Jaeger, e-mail: Martin.Jaeger@radboudumc.nl)]. Datasets can be made available under a data transfer agreement, and the data usage access is subject to local rules and regulations.
20	**Conflicts of Interest**	All authors should declare all potential conflicts of interest	24	The authors declare that they have no competing interests.

This checklist is copyrighted by the Equator Network under the Creative Commons Attribution 3.0 Unported (CC BY 3.0) license.
